# Comparative analysis of taxonomic, functional, and metabolic patterns of microbiomes from 14 full-scale biogas reactors by metagenomic sequencing and radioisotopic analysis

**DOI:** 10.1186/s13068-016-0465-6

**Published:** 2016-03-02

**Authors:** Gang Luo, Ioannis A. Fotidis, Irini Angelidaki

**Affiliations:** Shanghai Key Laboratory of Atmospheric Particle Pollution and Prevention (LAP3), Department of Environmental Science and Engineering, Fudan University, 200433 Shanghai, China; Department of Environmental Engineering, Technical University of Denmark, 2800 Kgs Lyngby, Denmark

**Keywords:** Biogas reactors, Metagenomic sequencing, Taxonomic patterns, Functional patterns, Methanogenic pathway

## Abstract

**Background:**

Biogas production is a very complex process due to the high complexity in diversity and interactions of the microorganisms mediating it, and only limited and diffuse knowledge exists about the variation of taxonomic and functional patterns of microbiomes across different biogas reactors, and their relationships with the metabolic patterns. The present study used metagenomic sequencing and radioisotopic analysis to assess the taxonomic, functional, and metabolic patterns of microbiomes from 14 full-scale biogas reactors operated under various conditions treating either sludge or manure.

**Results:**

The results from metagenomic analysis showed that the dominant methanogenic pathway revealed by radioisotopic analysis was not always correlated with the taxonomic and functional compositions. It was found by radioisotopic experiments that the aceticlastic methanogenic pathway was dominant, while metagenomics analysis showed higher relative abundance of hydrogenotrophic methanogens. Principal coordinates analysis showed the sludge-based samples were clearly distinct from the manure-based samples for both taxonomic and functional patterns, and canonical correspondence analysis showed that the both temperature and free ammonia were crucial environmental variables shaping the taxonomic and functional patterns. The study further the overall patterns of functional genes were strongly correlated with overall patterns of taxonomic composition across different biogas reactors.

**Conclusions:**

The discrepancy between the metabolic patterns determined by metagenomic analysis and metabolic pathways determined by radioisotopic analysis was found. Besides, a clear correlation between taxonomic and functional patterns was demonstrated for biogas reactors, and also the environmental factors that shaping both taxonomic and functional genes patterns were identified.

**Electronic supplementary material:**

The online version of this article (doi:10.1186/s13068-016-0465-6) contains supplementary material, which is available to authorized users.

## Background

Anaerobic digestion has been widely used for the treatment of organic wastes with simultaneous production of biogas. Biogas production from organic wastes includes four sequential metabolic steps (hydrolysis, fermentation, acetogenesis, and methanogenesis) and involves a number of different microorganisms (i.e., bacteria, archaea, fungi, and protozoa) [1]. The role and interaction of these microorganisms is very complex [2, 3], and it is vital to understand the taxonomic and functional dynamics of anaerobic digestion microbiomes at different settings of the biomethanation process, and their correlation with the metabolic pathway, in order to improve the process performance.

Several culture-independent molecular methods based on *16S rRNA* genes have been developed to investigate and characterize the microbiomes in biogas reactors [4–9]. The culture-independent methods include polymerase chain reaction (PCR)-denaturing gradient gel electrophoresis, PCR-terminal restriction fragment length polymorphism, PCR-cloning, and the recently developed PCR-high-throughput sequencing [10]. Numerous studies on microbial composition with respect to physical, chemical, and biological characteristics of biogas reactors have been published [1, 3, 11–14]. It is now known that the microbial composition is influenced by environmental variables such as temperature, feedstock, biogas reactor configurations, et al. [11, 12, 15–18]. Moreover, it is known that not only aceticlastic methanogens but also, depending on the operational conditions (e.g., ammonia concentration, et al.), hydrogenotrophic methanogens play a significant role in methanogenesis [19–21]. In addition, biogas reactors, operating at constant conditions (feedstock, temperature, et al.), have demonstrated an unprecedented level of stability with a unique community structure [3].

Our understanding of the microbial composition in biogas reactors has been increased greatly with the establishment of culture-independent molecular methods [3]. However, these molecular methods have several limitations, such as PCR bias [22], and lack of information about the functional genes of the microbiomes [23]. The ongoing development of high-throughput molecular tools and bioinformatics allows sequencing of the bulk DNA instead of only *16S rRNA* genes and thereby provides both taxonomic and functional information of microbiomes to an extent that was unimaginable even a few years ago [24]. It should be noted that traditional microbiological methodologies (e.g., isolation and cultivation of pure strains) have to be employed in order to study the physiology, metabolism, et al. for new isolates derived from biogas reactors, which could not be accomplished by metagenomic sequencing. Therefore, the combination of the new molecular technologies with traditional microbiological methodologies is necessary for future studies [16].

Metagenomic sequencing has been performed on different environments (agricultural soil, acid mine biofilm, sea, et al. [25]), and the first metagenomic analysis of biogas reactors was reported in 2008 [23]. Metagenomic studies on biogas reactors lacked an understanding of how functional genes encoded in their collective genomes, act across different biogas reactors, especially in correlation with different and/or changing environmental parameters (e.g., feedstock, temperature, process by-products such as ammonium, free ammonia nitrogen, or acids) [23, 26, 27]. In addition, previous studies estimated metabolic pathways (especially for methanogenesis) based on the corresponding functional genes by metagenomic analysis [26, 27]. Nevertheless, functional genes from metagenomic analysis only reflect the potential enzymes that could be synthesized by the microbes, and it is still not clear whether there is a direct correlation between metagenomic results and actual metabolic pathways taking place in the biogas reactors. Radioisotopic analysis and proteomic analysis have been widely used in the identification of the actual dominant methanogenic pathway in biogas reactors [9, 28].

Therefore, based on the above considerations, the aim of the present study was to conduct a detailed comparative analysis of both taxonomic and functional patterns of microbiomes from 14 different full-scale biogas reactors with a broad range of operational conditions by metagenomic sequencing and also to determine the predictability of actual metabolism by taxonomic and functional information. Specifically, the current study was designed to address the following questions: How is the relationship between the taxonomic and functional patterns of the microbiomes from biogas reactors and their correlation with the environmental variables? Is it possible to use the information on the taxonomic and functional compositions to predict the metabolic pathways (e.g., methanogenesis)?

## Results and discussion

### Overview of the metagenomics data

In total, around 400 million of 100 bp metagenomic reads for the 14 samples were obtained by paired-end sequencing (Additional file 1: Table S1), and the paired-end sequences were then joined to be joined reads with length around 170 bp (8,021,985–26,750,735 per sample). All the sequences were sub-sampled to 8,021,985 joined reads and then submitted to MG-RAST for further analysis. The percentages of identified *16S rRNA* genes, used for the taxonomic assignments [29], were between 0.042 and 0.132 % for all the samples, which were consistent with previous studies analyzing microbes in biogas reactors or in wastewater treatment bioreactors [5, 29]. The ratios of sequences annotated based on SEED subsystems by MG-RAST were between 19 and 35 % of the total sequences for all the samples (Additional file 1: Table S1). The annotated sequences levels were similar to those reported in previous studies, where metagenomic sequencing was used to characterize microbiomes in soil [30, 31] and in other highly diverse microbial habitats [32].

### Taxonomic classification

**Fig. 1:**
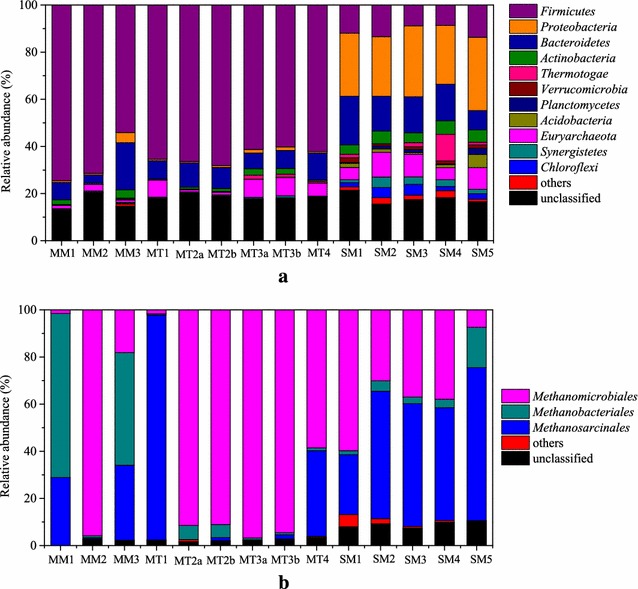
**a** Phylum level identification of all the sequences (Only relative abundances of identified Phylum higher than 1 % are listed, and all the other sequences are included in “others”); **b** Order level identification of all archaeal sequences (Only relative abundances of identified Order higher than 1 % in the archaeal sequences are listed, and all the other sequences are included in “others”)

In detail, six and eleven phyla (with relative abundance higher than 1 %) were identified in manure-based and sludge-based samples, respectively, demonstrating more diverse microbial communities for the sludge-based samples (Fig. 1). *Firmicutes* (54.8–75.8 %) and *Bacteroidetes* (3.5–20.2 %) were dominant in all the manure-based samples, while *Proteobacteria* (26.4–34.5 %) followed by *Firmicutes* (9.1–15.2 %) and *Bacteroidetes* (9.1–21.9 %) were dominant in all the sludge-based samples. The relative abundance of *Firmicutes* in manure-based samples (>50 %) was much higher than the sludge-based samples (<15 %). The dominance of *Firmicutes* in manure-based samples has also been reported in other studies [1, 10]. *Clostridia*, belonging to *Firmicutes*, were the most abundant class (>40 % of all the bacteria sequences) (Additional file 1: Table S2). For the manure-based samples, the relative abundances of *Firmicutes* and *Bacteroidetes* were more diverse for mesophilic samples compared with the thermophilic ones. The genus *Dechloromonas* [33], *Syntrophorhabdus* [34], and *Syntrophus* [35], which were chlorate-reducing, aromatic-degrading, and benzoate-degrading bacteria, were mainly found in sewage sludge-based samples, and it could be related to the corresponding pollutants contained in sewage sludge [36]. The cellulolytic bacteria *Halocella* [37] was mainly found in manure-based samples, and it could be due to the high lignocellulose contains in manure [38]. The presence of *Halocella* in biogas reactors, especially thermophilic reactors, was also reported previously [39].The observation of *Syntrophaceticus* mainly in samples MT2a, MT2b, MT3a, MT3b, and MT4 suggested syntrophic acetate-oxidizing occurred in the corresponding biogas reactors [40], which will be discussed later with the radioisotopic analysis data. In addition, it was found as expected that serial operated biogas reactors (MT2a and MT2b, MT3a, and MT3b) had similar microbial communities in both process steps (Additional file 1: Table S2). This is in accordance with our previous study where the microbial communities in the second reactor were found to be strongly correlated with the first reactor [41].

The relative abundance of sequences assigned to *Archaea* (i.e., methanogens) was low (from 1.2 to 11.4 %) for all samples (Additional file 1: Table S1), which was consistent with other studies [42]. Nevertheless, it does not necessarily mean that archaea had lower metabolic activity, since they might have higher transcriptional activity compared to bacteria, as has been recently demonstrated by comparing taxonomic results from *16S rRNA* genes, metagenomic, and metatranscriptomic analyses [43]. *Methanomicrobiales*, *Methanobacteriales*, and *Methanosarcinales* were the three dominant orders among all samples tested (Fig. 1b). Moreover, it should be noted that the dominant genus of the order *Methanosarcinales* was different for manure-based (*Methanosarcina*) and sludge-based samples (*Methanosaeta*) (Table 2), which could be related to the low VFA concentration in sludge-based samples compared to manure-based samples. *Methanosaeta* has higher affinity for acetate and grow better with lower acetate concentrations compared to *Methanosarcina* [44, 45].

For manure-based samples from thermophilic biogas reactors, MT1 sample was dominated by *Methanosarcinales* (Mainly *Methanosarcina* (around 90 %) as seen in Table 2, mediating aceticlastic methanogenesis and in some cases also hydrogenotrophic methanogenesis. [46]), while MT2a, MT2b, MT3a, and MT3b samples were dominated by hydrogenotrophic methanogens *Methanomicrobiales*. Differences between the relative abundances of the archaea orders among sludge-based samples were also observed. Sample SM1 had higher percentage of hydrogenotrophic methanogens *Methanomicrobiales*, while all the other samples (SM2–SM5) had higher percentages of *Methanosarcinales(Mainly Methanosaeta* (40–60 %) as seen in Table 2, mediating both aceticlastic methanogenesis*)*. Together, these observations suggest that there might be different dominant microorganisms mediating either hydrogenotrophic or aceticlastic methanogenesis for the samples from similar environments (e.g., manure-based samples from thermophilic biogas reactors and sludge-based samples from mesophilic biogas reactors).

### Functional classification

**Fig. 2:**
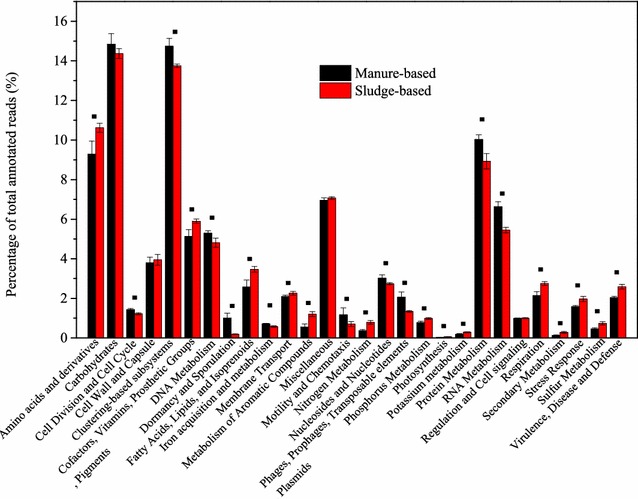
Average values of relative abundances of major categories of functional genes in the shotgun metagenomes obtained from manure-based and sludge-based samples. The *black square* indicate those categories with significantly different relative abundances in manure-based and sludge-based samples

The major functional categories for all the samples were those involved in metabolism of carbohydrates, clustering-based subsystems (containing such functions as proteosomes, ribosomes, and recombination-related clusters [12]), protein metabolism, amino acids, and derivatives (Additional file 1: Fig S1). Similar major functional genes were also found in the microbiomes of other ecosystems such as wastewater treatment system (activated sludge) [47], desert soil [30], and freshwater [48]. The comparison of the functional genes of sludge-based and manure-based samples (Fig. 2) demonstrated that 24 out of the total 25 major functional categories were found to be significantly different (p < 0.05 based on ANOVA analysis). There were no significant differences between manure-based and sludge-based samples for the functional category “Regulation and cell signaling.” There were several difference (e.g., nitrite, poly-3-hydroxybutyrate/poly-3-hydroxyvalerate, aromatic compounds, et al.) in the composition of manure and sludge which promoted the functionality variances observed. For example, the genes assigned into nitrogen metabolism and phosphorus metabolism had significantly higher abundances in sludge-based samples than manure-based samples, which was due to these specific processes taking place in wastewater treatment plants. In detail, the sludge-based samples were derived from wastewater treatment process and inevitably nitrate (from the aeration tank) [49] and poly-3-hydroxybutyrate/poly-3-hydroxyvalerate (accumulated in the waste-activated sludge for phosphorus removal) [50] were entering the biogas reactors. Furthermore, the sludge-based samples had more genes related to the metabolism of aromatic compounds. This can be explained by the higher abundance of aromatic compounds and other organic contaminants in the sludge originating from the wastewater, compared to manure-based digesters, which would promote establishment of microbes involved with metabolism of these compounds [51], and it was also consistent with the observation of aromatic–degrading *Syntrophorhabdus* mainly in sewage sludge samples (Additional file 1: Table S2) [34].

**Fig. 3:**
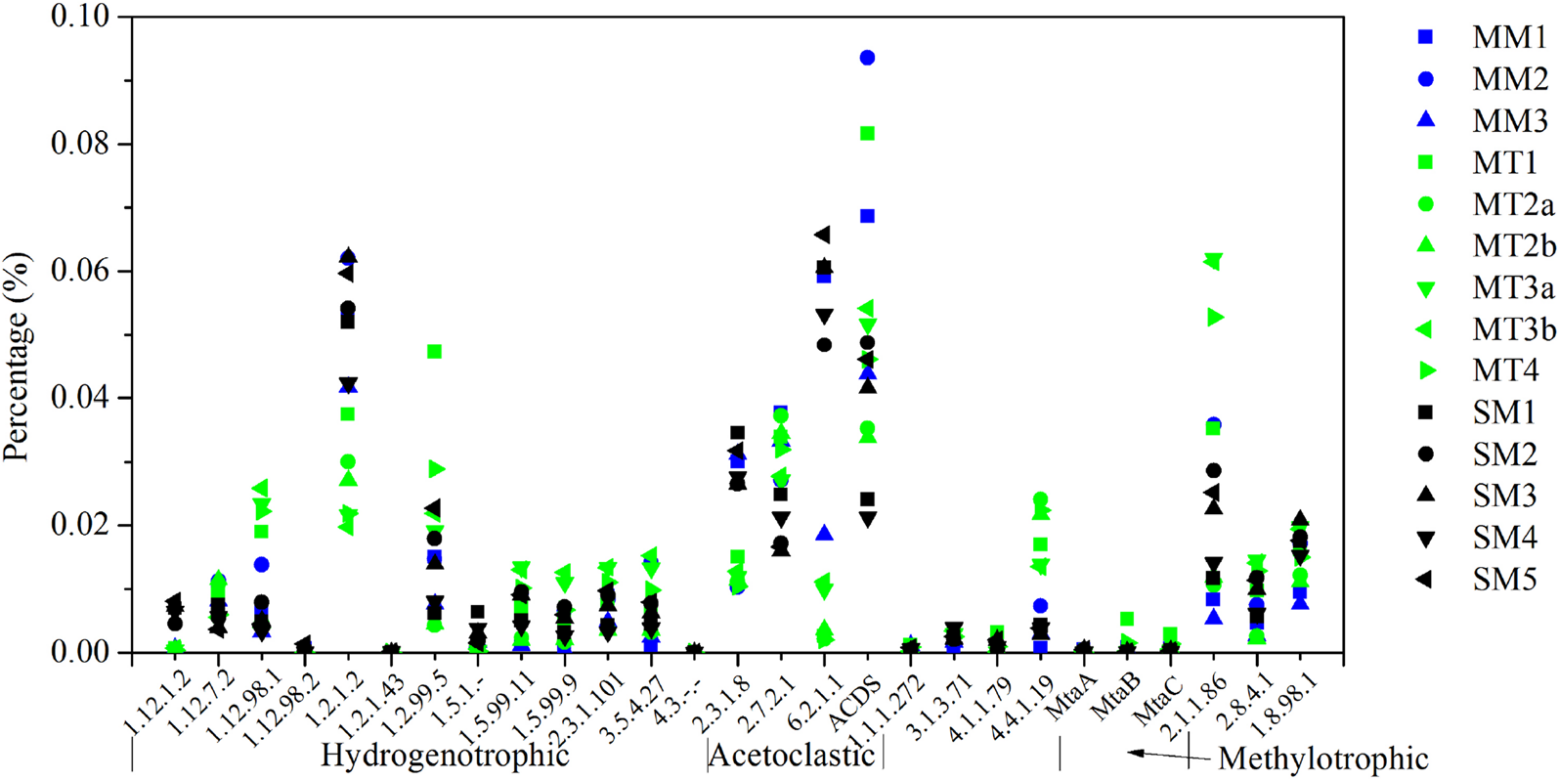
Genes involved in methanogenesis pathways from metagenomic datasets of the 14 samples

In order to make a more detailed analysis on the functional genes, the joined reads were annotated to metabolic pathways based on KEGG database. Protein and carbohydrate were the dominant compounds in manure and sewage sludge [41, 52], and therefore, the key genes relating to protein and carbohydrate anaerobic degradation were analyzed as shown in Additional file 1: Fig S2. The relative abundance of genes relating to cellulose and hemicellulose degradation were all higher in manure-based samples compared to sewage sludge-based samples, which was consistent with the high fiber content (lignocellulose materials) in manure [38]. For the genes relating to protein degradation, the relative abundances were similar for both sewage and manure-based samples. The genes relating to the metabolic pathways of methanogenesis [hydrogenotrophic (H_2_/CO_2_), acetoclastic (acetate), and methylotrophic (methanol)] were also analyzed, and the results are shown in Fig. 3 and Additional file 1: Table S3 [26, 53]. The dominant genes in hydrogenotrophic methanogenesis were formate dehydrogenase (EC: 1.2.1.2) and formylmethanofuran dehydrogenase (EC: 1.2.99.5), which were involved in the initial step of hydrogenotrophic pathway. The dominant genes in aceticlastic methanogenesis were acetyl-CoA synthetase (EC: 6.2.1.1) and acetyl-CoA decarbonylase/synthase complex (ACDS), and they were essential in the synthesis of acetyl-CoA from acetate. The most abundant genes were found to be related to hydrogenotrophic and aceticlastic methanogenic pathways in this study, and an interesting phenomenon was that hydrogenotrophic methanogens were absolutely dominant (the relative abundance higher than 95 % of archaea) in samples MM2, MT2a, MT2b, MT3a, and MT3b (Additional file 1: Fig S1). Additionally, genes encoding for aceticlastic methanogenesis is unique for this pathway and can therefore not be detected in hydrogenotrophic methanogens [54]. In fact, acetyl-CoA synthetase and acetyl-CoA decarbonylase/synthase complex also exist in bacteria and are involved in other metabolic pathways (acetate oxidation to produce H_2_ and CO_2_, homoacetogenesis to produce acetate, et al.) [55]. Thus, the claim that the abundance of different genes can be used to assess the methanogenic pathways in the biogas reactors [26, 27] seems not hold merit.

### Methanogenic pathway determined by radioisotopic analysis

It is generally assumed that aceticlastic methanogenesis is the dominant pathway when ^14^CO_2_/^14^CH_4_<1, while the hydrogenotrophic methanogenesis is the main pathway when ^14^CO_2_/^14^CH_4_>1 [28]. The results from radioisotopic analysis showed (Additional file 1: Fig S3) that all the sludge-based samples had ^14^CO_2_/^14^CH_4_<1, indicating the dominance of aceticlastic methanogenesis pathway. However, the dominant methanogenic pathway for all the manure-based samples except MT1 was hydrogenotrophic (^14^CO_2_/^14^CH_4_>1). The hydrogenotrophic pathway is coupled with syntrophic acetate oxidation that first converts acetate to H_2_/CO_2_ and then H_2_/CO_2_ is converted to methane by hydrogenotrophic methanogens. Syntrophic acetate oxidation is generally favored in the presence of inhibitors, particularly ammonium and volatile fatty acids [56], which may inhibit the aceticlastic methanogens. As shown in Table 1, all the manure-based samples had relatively higher ammonia and volatile fatty acids compared with sludge-based samples, which might be the reason for the dominance of hydrogenotrophic methanogenesis pathway in the manure-based samples. The dominance of syntrophic acetate-oxidizing *Syntrophaceticus* in MT2a, MT2b, MT3a, MT3b, and MT4 agreed well with the above results (Additional file 1: Table S2) [40]. VFA and ammonia had synergetic effect on the dominant methanogenic pathways as revealed by Lu et al. [21]. For MT1, the lower VFA and ammonia concentrations compared with the other manure-based samples might led to the dominance of aceticlastic methanogenesis pathway. It should be noted that *Methanosarcina* was dominant (90 %, Table 2) in MT1, while *Methanoculleus* was dominant in MT2a, MT2b, MT3a, MT3b, and MT4 (50–82 %, Table 2).

**Table 1:** Operating conditions of the full-scale biogas plants and related parameters of the samples

Sample name	Plant name	Main component in the feedstock	Reactor volume (m^3^)	HRT (days)	Biogas production (m^3^/m^3^/d)	Operating temperature (^o^C)	VFA (mM)	Ammonia-N (g/L)	pH	Free ammonia (g/L)
MM1	Nysted	Manure^a^	4800	21	2.85	37	6.32 ± 1.90	2.47 ± 0.05	7.83 ± 0.05	0.46 ± 0.07
MM2	Fangel	Manure	8000	32	2.25	40	6.93 ± 0.54	4.22 ± 0.01	8.18 ± 0.03	1.42 ± 0.09
MM3	Maabjerg	Manure	37,500	24	1.16	40	5.58 ± 0.20	2.63 ± 0.05	7.75 ± 0.07	0.42 ± 0.07
MT1	Sinding	Manure	750	22	3.2	52	1.95 ± 0.44	2.53 ± 0.04	7.82 ± 0.04	0.46 ± 0.04
MT2a	Blåhoj first step	Manure	1400	11	2.36	50	21.29 ± 8.54	2.96 ± 0.05	8.18 ± 0.08	0.99 ± 0.14
MT2b	Blåhoj second step	Manure	1400	11		52	10.74 ± 2.33	3.3 ± 0.05	8.36 ± 0.05	1.44 ± 0.10
MT3a	Lemvig first step	Manure	2400	15	2.86	52	4.49 ± 0.45	2.46 ± 0.04	8.10 ± 0.04	0.73 ± 0.05
MT3b	Lemvig second step	Manure	2400	3	0.74	52	1.05 ± 0.19	2.31 ± 0.07	8.10 ± 0.10	0.69 ± 0.13
MT4	Filskov	Manure	880	11	4.04	53	15.67 ± 0.17	2.38 ± 0.05	8.03 ± 0.06	0.63 ± 0.11
SM1	Maabjerg	Sewage sludge	9000	24	0.67	37	1.19 ± 0.13	0.47 ± 0.02	7.01 ± 0.04	0.02 ± 0.01
SM2	Luntoft	Sewage sludge	5000	30	3.3	37	0.65 ± 0.03	0.92 ± 0.03	7.11 ± 0.07	0.04 ± 0.01
SM3	Avedøre	Sewage sludge	6000	25	0.5	39	0.64 ± 0.07	0.53 ± 0.06	7.1 ± 0.03	0.02 ± 0.01
SM4	Helsinger	Sewage sludge	1400	19	0.5	37	0.60 ± 0.06	1.1 ± 0.02	7.33 ± 0.05	0.07 ± 0.01
SM5	Fakse	Sewage sludge	670	22	0.36	36	1.70 ± 1.29	1.24 ± 0.06	7.53 ± 0.09	0.13 ± 0.02

^a^ Manure from both cattle and swine. The co-fermentation feedstocks were industrial organic wastes, which accounted for around 10 %

**Table 2:** Genus level identification of the archaeal sequences (Only relative abundances of identified Class and Genus higher than 1 % were listed)

	MM1	MM2	MM3	MT1	MT2a	MT2b	MT3a	MT3b	MT4	SM1	SM2	SM3	SM4	SM5
*Methanospirillum*	0.00	0.00	0.00	0.00	0.00	0.00	0.00	0.00	0.00	9.72	6.81	9.72	10.28	2.03
*Methanoregula*	0.00	0.00	0.00	0.00	0.00	0.00	0.00	0.00	0.00	6.25	3.96	3.01	0.40	0.00
*Methanolinea*	0.00	0.00	0.00	0.00	0.00	0.00	0.00	0.00	0.00	16.67	5.49	8.33	1.19	2.25
*Methanosphaerula*	0.00	0.00	0.00	0.00	0.00	0.00	0.00	0.00	0.00	1.74	1.98	1.39	0.79	0.00
*Methanofollis*	0.00	0.25	0.00	0.10	6.84	4.83	5.09	5.08	3.46	1.39	0.88	0.69	0.00	0.00
*Methanomicrobium*	0.00	4.18	0.00	0.00	2.56	4.14	2.62	2.43	1.73	0.35	0.00	0.00	0.40	0.00
*Methanoculleus*	1.03	86.24	11.36	1.48	75.21	72.41	81.91	80.44	49.86	1.04	1.32	1.39	22.13	0.90
*Methanogenium*	0.52	0.00	4.55	0.00	0.00	0.00	0.05	0.00	0.00	0.00	0.00	0.00	0.00	0.00
*Methanosaeta*	0.00	0.00	3.41	0.00	0.00	0.00	0.00	0.00	0.09	22.57	45.71	48.15	40.71	59.46
*Methanosarcina*	27.32	0.25	23.86	90.41	0.00	0.69	0.14	1.74	34.28	0.00	0.00	0.00	0.00	0.23
*Methanobrevibacter*	61.86	0.49	34.09	0.20	5.13	3.45	0.57	0.38	0.82	0.69	1.54	0.46	2.37	3.60
*Methanobacterium*	1.55	0.25	4.55	0.20	0.00	2.07	0.00	0.08	0.27	0.35	0.88	1.39	0.40	11.49
*Methanosphaera*	6.19	0.00	6.82	0.10	0.85	0.00	0.14	0.30	0.09	0.00	1.10	0.46	0.00	0.68
Others	0.00	0.25	1.14	0.10	0.85	0.69	0.00	0.30	0.09	2.78	0.22	0.23	0.79	0.45
Unclassified	1.55	8.11	10.23	7.42	8.55	11.72	9.47	9.25	9.30	36.46	30.11	24.77	20.55	18.92

*Methanosarcinales* is the only order that could mediate methane production from acetate [57]. *Methanosaeta* (strict acetoclastic methanogens) and *Methanosarcina* (aceticlastic or hydrogenotrophic methanogens) were found to be the two main genus found in the order *Methanosarcinales* (Table 2). The correlation between the percentage of *Methanosarcinales* in total archaea sequences and the values of ^14^CO_2_/^14^CH_4_ from radioisotopic analysis is shown in Additional file 1: Fig S4. It is obvious that when the ratio of ^14^CO_2_/^14^CH_4_ was higher than two, *Methanosarcinales* were absent indicating an absolute dominance of hydrogenotrophic methanogens. Therefore, a ^14^CO_2_/^14^CH_4_ ratio threshold for absolute dominance of hydrogenotrophic methanogens in full-scale biogas reactors can be proposed. When the ^14^CO_2_/^14^CH_4_ ratio was between one and two, the percentage of *Methanosarcinales* was between 0–43 % which still showed the dominance of hydrogenotrophic methanogens. However, when the values of ^14^CO_2_/^14^CH_4_ were lower than one, the percentage of *Methanosarcinales* was above 43 %, with the exception of sample SM1, for which it was 23 %. It was further found that strict aceticlastic methanogens *Methanosaeta* was the predominant genus of *Methanosarcinales* for SM1 (22.6 % as shown in Table 2), while *Methanosarcina* was not found. It means that SM1 had dominant aceticlastic methanogenic pathway but at the same time lower relative abundance of aceticlastic methanogens. The reason could be that the analysis of relative abundance of *Methanosarcinales* was based on DNA, and it was not directly correlated with the activity of the relevant enzymes and the expression level of the genes. On contrary, radioisotopic analysis is relatively straightforward method to detect methanogenic activity by measuring the produced ^14^CH_4_ and ^14^CO_2_ from labeled acetate. Therefore, it seems more reasonable to use radioisotopic analysis instead of the relative abundance of the corresponding microorganisms to determine the dominant methanogenic pathway. Nonetheless, when *Methanosarcinales* were absent (or at very low abundance (<1 %), the dominant hydrogenotrophic methanogenesis pathway can also be predicted by the taxonomic composition. In order to get a clear metabolic patterns of the microbiomes in biogas reactors, metatranscriptomic and metaproteomic analysis unveiling the expression level of the genes and relevant enzymes, respectively, is necessary in the future studies [58].

### Variation of taxonomic and functional patterns and their correlations with environmental variables

**Fig. 4:**
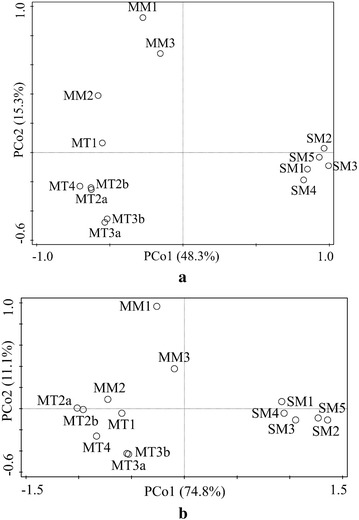
Principle coordinates analysis (PCoA) of the 14 samples based on both taxonomic (**a**) and functional (**b**) compositions

Principal coordinate analysis (PCoA) based on Bray-Curtis distance was used to visualize the distances and variations between samples (Fig. 4). The comparison of the microbial communities among the 14 samples (Fig. 4a) indicated that feedstock and temperature played important roles in the shaping of microbial communities in biogas reactors. The sludge-based biogas reactors harbored communities, clustered clearly apart from the manure-based biogas reactors’ communities. It was consistent with different organic components in sludge (microbial cells) and manure (lignocellulose) [38, 52]. In addition, there were clear differences between the samples from thermophilic and mesophilic manure-based reactors, which was expected since different microorganisms adapted to different temperatures [59]. However, mesophilic sample MM2 had a microbial community pattern close to those of thermophilic samples. This suggests that taxonomic patterns of mesophilic manure-based biogas reactors were more diverse compared with those of thermophilic manure-based and mesophilic sludge-based biogas reactors.

The comparison of the functional patterns among the 14 samples (Fig. 4b) showed that the sludge-based samples were clustered together and were well separated from manure-based samples. The functional pattern of sample MM2 (obtained from mesophilic manure-based biogas reactor) was closely clustered together with thermophilic manure-based biogas reactors. Further Procrustes analysis (Additional file 1: Fig S5) showed that generally functional patterns had significant correlation (p = 0.001) with taxonomic patterns by taking all the samples into consideration. The above results indicate that the functional patterns were identical to the taxonomic patterns. Therefore, one could predict the overall functional patterns from the taxonomic composition of the microbiomes, which was reported for the first time for biogas process, although similar conclusions were drawn from previous studies analyzing other ecosystems such as soil and gut microbiomes [60–62].

**Fig. 5:**
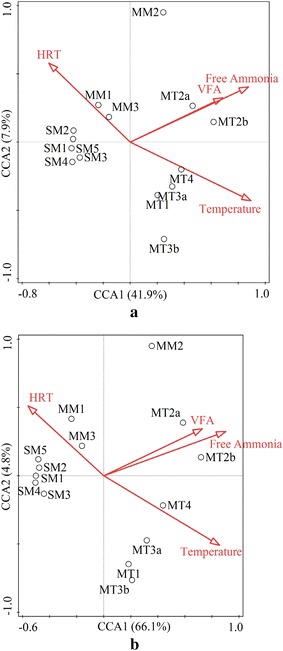
Canonical correspondence analysis (CCA) of the 14 samples based on the taxonomic compositions and environmental variables (**a**), and functional compositions and environmental variables (**b**)

Canonical correspondence analysis (CCA) was used to determine the most significant environmental variables to shape the taxonomic and functional patterns (Fig. 5). Four significant environmental variables (Temperature, VFA, HRT, and free ammonia) were chosen from Table 1 on the basis of VIFs <10 [63]. The CCA model explained 56.4 and 74.1 % of the total variance for taxonomic and functional patterns, respectively. The results showed that the selected environmental variables explained more for the variation of functional patterns compared to the variation of taxonomic patterns between samples. It should be noted that acetate accounted for more than 85 % of the total VFA as seen in Additional file 1: Table S4, and therefore the effect of VFA on taxonomic and functional patterns was mainly related with acetate. Besides, the distributions of samples in Fig. 5a, b were similar, which further suggests there is a correlation between taxonomic and functional patterns. Of the four selected environmental variables, temperature and free ammonia appears to be the most important environmental variables for both taxonomic and functional patterns. Although temperature and free ammonia were shown to affect the biogas production and microbial communities in biogas process [16, 20, 64], our study, for the first time, revealed that functional gene distributions were also determined by temperature and free ammonia.

## Conclusions

Our study made the first step to reveal the variation of taxonomic and functional patterns of microbiomes across different biogas reactors. Moreover, the relationship between the metabolic patterns determined by metagenomic analysis and metabolic pathways determined by radioisotopic analysis was elucidated. Although the microbial composition and functional genes in the complex biogas process can be assessed with the information from metagenomic sequencing, it was not possible to identify, with certainty, the true dominant methanogenic pathways of the biogas process using only the metagenomic sequencing assessment. In addition, a clear correlation between taxonomic and functional genes patterns was demonstrated for biogas reactors. The sludge-based samples were clearly distinct from the manure-based samples for both taxonomic and functional patterns based on PCoA analysis, and temperature and free ammonia were identified to be the important environmental variables shaping both taxonomic and functional patterns.

## Methods

### Sample collection

As shown in Table 1, 14 samples were collected from 12 Danish full-scale biogas plants operated either with manure and sewage sludge as main feedstocks and at different temperatures, hydraulic retention times, etc. It should be noted that two of the biogas plants (Blåhoj and Lemvig biogas plants) have two biogas reactors running in series, and thus samples (samples names: MT2a, MT2b, MT3a, and MT3b) were collected from both steps of the series process. All the biogas plants had been running for more than 2 years under similar operational conditions. All the biogas plants reported normal operational conditions at the time of sampling, and no major changes had occurred prior to sampling, which ensured the representativeness of the samples. The samples for the microbial analysis were collected in sterile tubes (15 mL) and were frozen immediately in a cooler with dry ice. The samples for chemical analysis were collected in 0.5 L bottles and put in a cooler box with ice, while the samples for biological activities test were kept at ambient temperature. All biogas reactors had sampling points in the effluent lines close to the reactors ensuring good representative samples of the reactor biomass. The sampling valve was opened for 5 min before sample acquisition to flush the sampling valve and tube. After sampling, the samples were transported to the laboratory within 1 day. The parameters including VFA and ammonia were analyzed in our lab, while all the other parameters were obtained from the records of the specific biogas plant.

### DNA extraction and metagenomic analysis

Total genomic DNA of each collected sample was extracted using QIAamp DNA Stool Mini Kit (QIAGEN, 51504) according to the manufacturer’s instructions. Libraries with insert size of 180 bp were constructed according to the manufacturer’s instructions (Illumina) for the samples. Sequencing was conducted using Illumina Hiseq 2000 platform by applying 101 bp paired-end strategy.

Sequence reads were initially filtered to remove those containing bases with quality score lower than 30 and containing one or more uncalled bases [5]. All the pair-end reads of each dataset were joined to decrease the sequencing errors, and the reads that did not overlap were removed. The joined reads had an average length around 170 bp. To obtain a quantitative picture of the taxonomic and functional patterns, all the joined reads after sub-sampled to the same sequencing depth were uploaded to MG-RAST (Rapid Annotation using Subsystems Technology for Metagenomes) for downstream analyses with the project ID 6474 [65]. For taxonomic analysis, the *16S rRNA* gene sequences with hits were extracted from the results of BLAST analysis against the Ribosomal Database Project (RDP) database with an E-value cutoff <10^−5^. Only 16S rRNA fragments with at least 90 % assignment confidence were considered. The *16S rRNA* gene sequences were then submitted to the RDP database (http://rdp.cme.msu.edu/) for classification with 50 % confidence. The 50 % confidence is recommended by RDP and used in many studies [6, 66]. For functional analysis, SEED subsystems and KEGG annotation were used to assign joined reads to different functional groups (SEED) or metabolic pathways (KEGG) in MG-RAST using the parameters of E-value cutoff 10^−5^ and minimum alignment length 50 bp, and only the annotated sequences were used for further analysis [30, 31]. Principal coordinates analysis (PCoA) and canonical correspondence analysis (CCA) were conducted by Canoco 5.0 to explore the taxonomic and functional relationships between the samples and also to identify the key environmental variables shaping the taxonomic and functional gene compositions. Procrustes analysis was performed by R (v.2.13.1; http://www.r-project.org/) with packages VEGAN. The relative abundance of *16S rRNA* gene reads assigned to Genus by RDP and joined reads assigned to Subsystem Level three by MG-RAST were used for both PCoA and CCA analysis, which should yield a more conservative estimate of the distance between the samples compared to the reads assigned to species and individual genes. PCoA is the most commonly used dimensionality reduction techniques in microbial ecology to visualize the different patterns of various samples [13]. CCA was used to explore the relationship between different patterns and environmental variables [14].

### Radioisotopic analysis

The methanogenic pathway of acetate degradation was determined by measuring the production of ^14^CH_4_ and ^14^CO_2_ from acetate labeled in the methyl group (C-2) [28]. In the radioisotopic experiments, 118-mL glass batch reactors were used and 40 mL of inoculum was dispensed anaerobically under a N_2_/CO_2_ (80/20 %) headspace. For each of the 14 samples derived from the full-scale biogas reactors, the following batch reactors were included: (a) three reactors (n = 3) with inoculum only for estimation of residual methane production and (b) three reactors (n = 3) containing 47.58 ± 5.49 KBq L^−1^ [2-^14^C] sodium acetate (Amersham Pharmacia Biotech, England) for identification of the methanogenic pathway (calculation of ^14^CO_2_/^14^CH_4_ ratio). Afterward, the batch reactors were closed with butyl rubber stoppers, sealed with aluminum caps, and then incubated in a thermostatic incubator at 37 or 55 °C based on their original temperature of the biogas reactors (Table 1), until methane production ceased (around 1 month). Then, bottles containing labeled acetate were acidified (final pH = 0.95 ± 0.1) with 7.2 M HCl, resulting in the conversion of dissolved bicarbonate to CO_2_. The liquid and headspace of each bottle was sparged with approximately 2 L of O_2_, and the labeled ^14^CO_2_ was trapped with a carbon dioxide absorber for liquid scintillation counting (10 mL of Carbosorb^R^-E; Perkin-Elmer Company). Subsequently, the labeled ^14^CH_4_ was combusted to ^14^CO_2_ in a tube furnace above 800 °C, and the ^14^CO_2_ generated in the furnace was then trapped in 10 mL of Carbosorb^R^-E. For counting, the 10 mL of Carbosorb^R^-E were mixed with 10 mL Permaflour RE (Perkin-Elmer Company) scintillation fluid. All radioactivity measurements were performed using a liquid scintillation counter (Tri-Carb 1600; Perkin-Elmer Company).

### Analytical methods

Total ammonia was determined by the Kjeldahl method according to American Public Health Association’s Standard Methods [67]. The concentrations of acetate, propionate, iso-butyrate, butyrate, iso-valerate, and valerate were determined by gas chromatograph (GC) (Hewlett Packard, HP5890 series II) equipped with a flame ionization detector and HP FFAP column (30 m × 0.53 mm × 1.0 μm). CH_4_ was analyzed by GC-TCD fitted with parallel column of 1.1 m × 3/16 "Molsieve 137 and 0.7 m × 1/4" chromosorb 108. Detailed information about the operational conditions of above GC was described previously [68]. All analyses were made in triplicate, and the averages are presented along with the corresponding standard deviations (SD) calculated from the analyses. An analysis of variance (ANOVA) was used to test the significance of results, and p < 0.05 was considered to be statistically significant.

## Additional file

10.1186/s13068-016-0465-6 Table S1 Summary of the metagenomic sequencing data; Table S2 Taxonomic classification of the bacterial sequences (Only relative abundances of identified Class and Genus higher than 1 % were listed); Table S3 Relative abundance of each gene involved in methanogenesis pathways from metagenomic datasets of the 14 samples; Table S4 Individual VFAs in each sample; Figure S1 Relative abundances of major categories of functional genes in the shotgun metagenomes obtained from the 14 samples; Figure S2 Genes involved in carbohydrate and protein pathways from metagenomic datasets of the 14 samples; Figure S3 The ratio of 14CO_2_/14CH_4 _of the 14 samples; Figure S4 Correlation between the ratio of 14CO_2_/14CH_4_ and percentage of Methanosarcinales; Figure S5 Procrustes analyses of taxonomic and functional patterns.

## Abbreviations

HRT: hydraulic retention time
VFA: volatile fatty acids
PCoA: principal coordinates analysis
CCA: canonical correspondence analysis
