# Supplementary material for: Comparative analysis of taxonomic, functional, and metabolic patterns of microbiomes from 14 full-scale biogas reactors by metagenomic sequencing and radioisotopic analysis
Source: Biotechnol Biofuels. 2016 Mar 2;9:51. doi: 10.1186/s13068-016-0465-6 (PMC4776419; doi:10.1186/s13068-016-0465-6)
Supplement: Supplementary file 1 — 10.1186/s13068-016-0465-6 Table S1 Summary of the metagenomic sequencing data; Table S2 Taxonomic classification of the bacterial sequences (Only relative abundances of identified Class and Genus higher than 1 % were listed); Table S3 Relative abundance of each gene involved in methanogenesis pathways from metagenomic datasets of the 14 samples; Table S4 Individual VFAs in each sample; Figure S1 Relative abundances of major categories of functional genes in the shotgun metagenomes obtained from the 14 samples; Figure S2 Genes involved in carbohydrate and protein pathways from metagenomic datasets of the 14 samples; Figure S3 The ratio of 14CO2/14CH4 of the 14 samples; Figure S4 Correlation between the ratio of 14CO2/14CH4 and percentage of Methanosarcinales; Figure S5 Procrustes analyses of taxonomic and functional patterns. [file 13068_2016_465_MOESM1_ESM.docx]

Supporting information:

**Comparative analysis of taxonomic, functional and metabolic patterns of microbiomes from 14 full-scale biogas reactors by metagenomic sequencing and radioisotopic analysis**

Gang Luo^1^, Ioannis A. Fotidis^2^, Irini Angelidaki^2*^

^1^ Shanghai Key Laboratory of Atmospheric Particle Pollution and Prevention (LAP3), Department of Environmental Science and Engineering, Fudan University, 200433, Shanghai, China

^2^ Department of Environmental Engineering, Technical University of Denmark, DK-2800, Kgs Lyngby, Denmark

Email:

Gang Luo: [gangl@fudan.edu.cn](mailto:gangl@fudan.edu.cn)

Ioannis A. Fotidis: ioanf@env.dtu.dk

Irini Angelidaki: [iria@env.dtu.dk](mailto:iria@env.dtu.dk%20)

*Corresponding author:

Irini Angelidaki: [iria@env.dtu.dk](mailto:iria@env.dtu.dk%20)

Table S1 Summary of the metagenomic sequencing data

| Sample name | Reads  (100bp) | Tags (~170bp) | Assigned protein (%) | Percentage of identified 16S rRNA gene (%) | Percentage of bacteria in the identified 16S rRNA gene (%) | Percentage of archaea in the identified 16S rRNA gene (%) |
| --- | --- | --- | --- | --- | --- | --- |
| MM1 | 12,467,700*2 | 10,752,111 | 24.2 | 0.132 | 98.3 | 1.7 |
| MM2 | 13,186,150*2 | 11,782,875 | 21.8 | 0.127 | 97.2 | 2.8 |
| MM3 | 9626100*2 | 8,021,985 | 32.8 | 0.116 | 98.5 | 1.5 |
| MT1 | 12,611,150*2 | 11,014,099 | 23.1 | 0.126 | 92.5 | 7.5 |
| MT2a | 12,821,750*2 | 9,064,863 | 20.1 | 0.134 | 98.8 | 1.2 |
| MT2b | 13,163,550*2 | 9,977,217 | 20.9 | 0.128 | 98.8 | 1.2 |
| MT3a | 30,014,200*2 | 26,750,735 | 21.8 | 0.103 | 92.1 | 7.9 |
| MT3b | 18,662,400*2 | 16,452,526 | 22 | 0.103 | 92.9 | 7.1 |
| MT4 | 16,272,400*2 | 14,852,689 | 19.1 | 0.132 | 94.2 | 5.8 |
| SM1 | 15,306,650*2 | 12,779,969 | 30.3 | 0.043 | 93.8 | 6.2 |
| SM2 | 10,569,300*2 | 9,369,214 | 33.7 | 0.046 | 88.6 | 11.4 |
| SM3 | 11,968,650*2 | 10,656,756 | 34.8 | 0.042 | 89.2 | 10.8 |
| SM4 | 10,761,650*2 | 9,199,240 | 33.6 | 0.053 | 93.9 | 6.1 |
| SM5 | 12,027,200*2 | 9,804,037 | 34.4 | 0.049 | 89.5 | 10.5 |

Table S2 Taxonomic calssification of the bacterial sequences (Only relative abundances of identified Class and Genus higher than 1% were listed)

|  | MM1 | MM2 | MM3 | MT1 | MT2a | MT2b | MT3a | MT3b | | MT4 | SM1 | SM2 | SM3 | SM4 | SM5 |
| --- | --- | --- | --- | --- | --- | --- | --- | --- | --- | --- | --- | --- | --- | --- | --- |
| **Phylum** |  |  |  |  |  |  |  |  |  | |  |  |  |  |  |
| *Firmicutes* | 75.76 | 73.42 | 54.83 | 70.73 | 67.09 | 68.83 | 66.41 | 65.44 | 65.92 | | 12.76 | 15.19 | 9.84 | 9.19 | 15.21 |
| *Proteobacteria* | 0.75 | 0.76 | 4.38 | 0.66 | 0.65 | 0.92 | 1.67 | 1.63 | 0.63 | | 28.29 | 28.38 | 33.60 | 26.42 | 34.55 |
| *Bacteroidetes* | 7.46 | 3.54 | 20.22 | 8.39 | 10.34 | 9.01 | 7.21 | 8.40 | 12.14 | | 21.93 | 16.66 | 17.07 | 16.41 | 9.08 |
| *Verrucomicrobia* | 0.31 | 0.03 | 0.44 | 0.01 | 0.00 | 0.01 | 0.02 | 0.04 | 0.01 | | 2.15 | 0.95 | 1.24 | 1.41 | 1.60 |
| *Planctomycetes* | 0.08 | 0.00 | 0.26 | 0.01 | 0.01 | 0.02 | 0.04 | 0.07 | 0.01 | | 0.35 | 1.42 | 1.62 | 0.33 | 2.96 |
| *Actinobacteria* | 2.06 | 0.59 | 3.55 | 0.30 | 0.86 | 1.29 | 3.00 | 2.60 | 0.60 | | 4.17 | 5.99 | 4.67 | 6.05 | 5.83 |
| *Acidobacteria* | 0.01 | 0.00 | 0.20 | 0.01 | 0.01 | 0.00 | 0.02 | 0.01 | 0.00 | | 1.89 | 1.63 | 0.75 | 1.37 | 6.18 |
| *Thermotogae* | 0.00 | 0.00 | 0.00 | 0.13 | 0.28 | 0.32 | 1.84 | 1.36 | 0.75 | | 1.54 | 0.16 | 1.91 | 11.99 | 1.23 |
| *Chloroflexi* | 0.03 | 0.01 | 0.15 | 0.03 | 0.01 | 0.00 | 0.04 | 0.08 | 0.01 | | 2.01 | 4.81 | 5.12 | 1.86 | 2.55 |
| *Synergistetes* | 0.40 | 0.46 | 0.52 | 0.38 | 0.37 | 0.39 | 0.63 | 0.93 | 0.22 | | 1.29 | 4.99 | 3.60 | 3.03 | 2.06 |
| *Spirochaetes* | 0.09 | 0.01 | 0.46 | 0.09 | 0.12 | 0.12 | 0.17 | 0.18 | 0.06 | | 0.59 | 0.45 | 0.89 | 2.19 | 0.37 |
| *other* | 0.06 | 0.23 | 0.47 | 0.03 | 0.02 | 0.03 | 0.13 | 0.22 | 0.05 | | 0.80 | 2.63 | 1.12 | 0.85 | 0.83 |
| *unclassified* | 12.99 | 20.94 | 14.53 | 19.24 | 20.25 | 19.07 | 18.81 | 19.04 | 19.61 | | 22.24 | 16.76 | 18.56 | 18.92 | 17.55 |
| **Class** |  |  |  |  |  |  |  |  |  | |  |  |  |  |  |
| *Sphingobacteria* | 0.16 | 0.11 | 1.42 | 0.42 | 0.58 | 0.52 | 0.44 | 0.38 | 0.73 | | 6.94 | 3.31 | 2.51 | 3.32 | 2.36 |
| *Bacteroidia* | 5.25 | 2.10 | 10.91 | 2.61 | 1.85 | 1.46 | 1.59 | 2.07 | 2.13 | | 3.00 | 5.52 | 4.72 | 4.27 | 1.44 |
| *Flavobacteria* | 0.38 | 0.08 | 1.88 | 0.34 | 0.33 | 0.30 | 0.26 | 0.35 | 0.40 | | 3.53 | 2.00 | 1.59 | 1.60 | 1.25 |
| *Subdivision3* | 0.00 | 0.00 | 0.01 | 0.00 | 0.00 | 0.00 | 0.00 | 0.00 | 0.00 | | 1.52 | 0.45 | 0.35 | 1.28 | 0.65 |
| *Deltaproteobacteria* | 0.08 | 0.22 | 0.17 | 0.04 | 0.05 | 0.06 | 0.05 | 0.03 | 0.03 | | 11.80 | 3.78 | 4.80 | 6.00 | 4.86 |
| *Gammaproteobacteria* | 0.18 | 0.01 | 1.19 | 0.22 | 0.07 | 0.07 | 0.22 | 0.18 | 0.11 | | 2.48 | 5.28 | 6.91 | 2.47 | 4.65 |
| *Betaproteobacteria* | 0.11 | 0.01 | 0.92 | 0.02 | 0.05 | 0.08 | 0.57 | 0.50 | 0.09 | | 7.39 | 9.56 | 12.20 | 11.73 | 10.16 |
| *Alphaproteobacteria* | 0.20 | 0.08 | 1.04 | 0.03 | 0.24 | 0.40 | 0.52 | 0.57 | 0.15 | | 2.20 | 6.59 | 6.64 | 2.67 | 11.97 |
| *Planctomycetacia* | 0.07 | 0.00 | 0.26 | 0.01 | 0.01 | 0.02 | 0.04 | 0.07 | 0.01 | | 0.25 | 1.42 | 1.62 | 0.30 | 2.89 |
| *Actinobacteria* | 2.06 | 0.59 | 3.55 | 0.30 | 0.86 | 1.29 | 3.00 | 2.60 | 0.60 | | 4.17 | 5.99 | 4.67 | 6.05 | 5.83 |
| *Clostridia* | 63.14 | 54.12 | 41.79 | 48.21 | 42.30 | 43.93 | 41.59 | 41.25 | 42.43 | | 9.58 | 10.54 | 6.29 | 6.76 | 11.72 |
| *Bacilli* | 2.00 | 1.46 | 2.07 | 2.27 | 2.06 | 2.03 | 3.87 | 2.94 | 3.01 | | 0.18 | 1.34 | 0.80 | 0.43 | 0.81 |
| *Negativicutes* | 0.34 | 1.35 | 0.62 | 1.45 | 1.58 | 1.48 | 1.06 | 1.19 | 0.96 | | 0.27 | 0.32 | 0.22 | 0.33 | 0.30 |
| *Thermotogae* | 0.00 | 0.00 | 0.00 | 0.13 | 0.28 | 0.32 | 1.84 | 1.36 | 0.75 | | 1.54 | 0.16 | 1.91 | 11.99 | 1.23 |
| *Anaerolineae* | 0.01 | 0.00 | 0.07 | 0.02 | 0.00 | 0.00 | 0.02 | 0.02 | 0.00 | | 1.70 | 3.57 | 4.08 | 1.15 | 1.46 |
| *Synergistia* | 0.40 | 0.46 | 0.52 | 0.38 | 0.37 | 0.39 | 0.63 | 0.93 | 0.22 | | 1.29 | 4.99 | 3.60 | 3.03 | 2.06 |
| *other* | 1.43 | 0.83 | 2.05 | 0.45 | 0.92 | 1.35 | 0.55 | 0.71 | 0.84 | | 3.73 | 5.39 | 4.08 | 5.20 | 7.46 |
| *unclassified* | 24.20 | 38.59 | 31.53 | 43.11 | 48.46 | 46.29 | 43.75 | 44.86 | 47.54 | | 38.41 | 29.80 | 33.03 | 31.43 | 28.90 |
| **Order** |  |  |  |  |  |  |  |  |  | |  |  |  |  |  |
| *Sphingobacteriales* | 0.16 | 0.11 | 1.42 | 0.42 | 0.58 | 0.52 | 0.44 | 0.38 | 0.73 | | 6.94 | 3.31 | 2.51 | 3.32 | 2.36 |
| *Bacteroidales* | 5.25 | 2.10 | 10.91 | 2.61 | 1.85 | 1.46 | 1.59 | 2.07 | 2.13 | | 3.00 | 5.52 | 4.72 | 4.27 | 1.44 |
| *Flavobacteriales* | 0.38 | 0.08 | 1.88 | 0.34 | 0.33 | 0.30 | 0.26 | 0.35 | 0.40 | | 3.53 | 2.00 | 1.59 | 1.60 | 1.25 |
| *Syntrophobacterales* | 0.00 | 0.00 | 0.00 | 0.00 | 0.00 | 0.00 | 0.00 | 0.00 | 0.01 | | 6.81 | 1.42 | 1.52 | 2.90 | 2.18 |
| *Xanthomonadales* | 0.01 | 0.00 | 0.33 | 0.02 | 0.02 | 0.03 | 0.11 | 0.14 | 0.01 | | 1.40 | 2.89 | 1.91 | 1.30 | 2.66 |
| *Rhodocyclales* | 0.01 | 0.00 | 0.56 | 0.00 | 0.00 | 0.02 | 0.39 | 0.30 | 0.02 | | 3.73 | 2.76 | 3.31 | 3.94 | 3.61 |
| *Burkholderiales* | 0.09 | 0.01 | 0.26 | 0.02 | 0.03 | 0.06 | 0.11 | 0.14 | 0.06 | | 2.48 | 5.65 | 6.68 | 6.55 | 5.00 |
| *Sphingomonadales* | 0.01 | 0.00 | 0.03 | 0.00 | 0.00 | 0.02 | 0.02 | 0.03 | 0.01 | | 0.59 | 1.08 | 0.99 | 0.26 | 1.50 |
| *Rhizobiales* | 0.11 | 0.05 | 0.63 | 0.02 | 0.10 | 0.15 | 0.22 | 0.24 | 0.03 | | 0.64 | 2.50 | 2.88 | 1.15 | 6.90 |
| *Rhodobacterales* | 0.06 | 0.02 | 0.14 | 0.01 | 0.12 | 0.21 | 0.18 | 0.17 | 0.10 | | 0.55 | 2.15 | 1.57 | 0.50 | 1.32 |
| *Actinomycetales* | 0.77 | 0.41 | 3.00 | 0.20 | 0.36 | 0.64 | 2.79 | 2.40 | 0.26 | | 3.20 | 3.89 | 2.44 | 4.20 | 2.87 |
| *Clostridiales* | 59.04 | 41.30 | 36.84 | 35.48 | 25.22 | 26.65 | 28.08 | 28.74 | 28.31 | | 9.34 | 10.04 | 5.86 | 6.39 | 11.16 |
| *Thermoanaerobacterales* | 0.11 | 0.21 | 0.23 | 0.28 | 2.84 | 2.14 | 2.10 | 1.88 | 2.08 | | 0.00 | 0.00 | 0.00 | 0.00 | 0.02 |
| *Halanaerobiales* | 0.00 | 1.41 | 0.03 | 1.97 | 3.29 | 4.11 | 1.53 | 0.60 | 2.96 | | 0.00 | 0.05 | 0.00 | 0.00 | 0.00 |
| *Lactobacillales* | 1.70 | 0.50 | 1.48 | 0.56 | 0.26 | 0.38 | 1.58 | 0.87 | 0.85 | | 0.14 | 1.02 | 0.37 | 0.41 | 0.02 |
| *Bacillales* | 0.21 | 0.86 | 0.41 | 1.30 | 1.32 | 1.27 | 1.84 | 1.67 | 1.74 | | 0.04 | 0.29 | 0.40 | 0.00 | 0.76 |
| *Selenomonadales* | 0.34 | 1.35 | 0.62 | 1.45 | 1.58 | 1.48 | 1.06 | 1.19 | 0.96 | | 0.27 | 0.32 | 0.22 | 0.33 | 0.30 |
| *Erysipelotrichales* | 1.01 | 0.54 | 0.70 | 0.33 | 0.77 | 1.19 | 0.27 | 0.31 | 0.73 | | 0.14 | 0.08 | 0.12 | 0.07 | 0.05 |
| *Thermotogales* | 0.00 | 0.00 | 0.00 | 0.13 | 0.28 | 0.32 | 1.84 | 1.36 | 0.75 | | 1.54 | 0.16 | 1.91 | 11.99 | 1.23 |
| *Anaerolineales* | 0.01 | 0.00 | 0.07 | 0.02 | 0.00 | 0.00 | 0.02 | 0.02 | 0.00 | | 1.70 | 3.57 | 4.08 | 1.15 | 1.46 |
| *Synergistales* | 0.40 | 0.46 | 0.52 | 0.38 | 0.37 | 0.39 | 0.63 | 0.93 | 0.22 | | 1.29 | 4.99 | 3.60 | 3.03 | 2.06 |
| *other* | 1.81 | 0.78 | 2.74 | 1.06 | 1.45 | 1.55 | 1.39 | 1.31 | 1.12 | | 4.92 | 8.88 | 10.41 | 6.48 | 9.31 |
| *unclassified* | 28.50 | 49.80 | 37.20 | 53.42 | 59.22 | 57.12 | 53.54 | 54.90 | 56.53 | | 47.76 | 37.44 | 42.89 | 40.16 | 42.53 |
| **Genus** |  |  |  |  |  |  |  |  |  | |  |  |  |  |  |
| *Subdivision3_genera_incertae_sedis* | 0.00 | 0.00 | 0.01 | 0.00 | 0.00 | 0.00 | 0.00 | 0.00 | 0.00 | | 1.52 | 0.45 | 0.35 | 1.28 | 0.65 |
| *Thermovirga* | 0.02 | 0.01 | 0.05 | 0.01 | 0.00 | 0.02 | 0.00 | 0.00 | 0.00 | | 0.41 | 2.05 | 1.07 | 1.39 | 0.21 |
| *Anaerolinea* | 0.00 | 0.00 | 0.00 | 0.01 | 0.00 | 0.00 | 0.01 | 0.01 | 0.00 | | 0.41 | 1.29 | 0.89 | 0.09 | 0.35 |
| *Fervidobacterium* | 0.00 | 0.00 | 0.00 | 0.00 | 0.00 | 0.01 | 0.00 | 0.00 | 0.00 | | 0.00 | 0.00 | 0.00 | 9.90 | 0.02 |
| *Treponema* | 0.07 | 0.01 | 0.29 | 0.04 | 0.02 | 0.06 | 0.02 | 0.01 | 0.04 | | 0.33 | 0.21 | 0.32 | 1.11 | 0.25 |
| *Halocella* | 0.00 | 1.03 | 0.03 | 1.60 | 2.50 | 3.05 | 1.10 | 0.49 | 2.03 | | 0.00 | 0.03 | 0.00 | 0.00 | 0.00 |
| *Syntrophaceticus* | 0.01 | 0.10 | 0.01 | 0.02 | 1.64 | 1.11 | 1.18 | 1.07 | 1.15 | | 0.00 | 0.00 | 0.00 | 0.00 | 0.00 |
| *Lactobacillus* | 0.58 | 0.19 | 0.44 | 0.26 | 0.05 | 0.09 | 1.18 | 0.67 | 0.34 | | 0.00 | 0.03 | 0.00 | 0.00 | 0.00 |
| *Alkaliphilus* | 1.36 | 0.54 | 0.49 | 0.00 | 0.01 | 0.02 | 0.01 | 0.01 | 0.02 | | 0.02 | 0.00 | 0.00 | 0.00 | 0.02 |
| *Tissierella* | 0.41 | 5.67 | 0.47 | 0.13 | 0.22 | 0.28 | 0.11 | 0.09 | 0.29 | | 0.00 | 0.03 | 0.00 | 0.02 | 0.51 |
| *Peptoniphilus* | 0.23 | 1.01 | 0.04 | 0.02 | 0.03 | 0.04 | 0.14 | 0.15 | 0.09 | | 0.00 | 0.00 | 0.00 | 0.00 | 0.00 |
| *Tepidimicrobium* | 0.07 | 0.22 | 0.07 | 0.20 | 0.32 | 0.32 | 1.23 | 0.98 | 0.48 | | 0.00 | 0.00 | 0.00 | 0.00 | 0.02 |
| *Clostridium XI* | 3.11 | 1.72 | 1.24 | 0.72 | 0.67 | 0.54 | 0.51 | 0.40 | 1.17 | | 0.20 | 0.11 | 0.10 | 0.41 | 0.07 |
| *Gracilibacter* | 0.00 | 0.00 | 0.03 | 0.00 | 0.01 | 0.00 | 0.00 | 0.00 | 0.00 | | 2.30 | 0.03 | 0.32 | 0.56 | 1.34 |
| *Syntrophomonas* | 0.73 | 2.87 | 1.30 | 5.71 | 0.97 | 1.10 | 1.10 | 1.27 | 2.44 | | 1.56 | 0.74 | 0.50 | 0.59 | 0.39 |
| *Clostridium sensu stricto* | 3.80 | 3.25 | 1.94 | 0.85 | 0.24 | 0.28 | 0.19 | 0.17 | 0.27 | | 0.12 | 0.08 | 0.00 | 0.28 | 0.14 |
| *Clostridium III* | 1.82 | 1.15 | 1.34 | 3.26 | 1.99 | 2.23 | 1.81 | 1.63 | 2.05 | | 0.23 | 0.13 | 0.67 | 0.82 | 0.19 |
| *Saccharofermentans* | 3.86 | 0.03 | 0.73 | 0.02 | 0.02 | 0.07 | 0.02 | 0.02 | 0.03 | | 0.06 | 1.45 | 0.15 | 0.15 | 0.12 |
| *Acidovorax* | 0.00 | 0.00 | 0.00 | 0.00 | 0.00 | 0.00 | 0.00 | 0.01 | 0.00 | | 0.18 | 1.29 | 1.14 | 0.82 | 0.51 |
| *Thauera* | 0.01 | 0.00 | 0.48 | 0.00 | 0.00 | 0.01 | 0.35 | 0.29 | 0.00 | | 0.76 | 0.76 | 1.32 | 0.20 | 0.19 |
| *Dechloromonas* | 0.00 | 0.00 | 0.00 | 0.00 | 0.00 | 0.00 | 0.00 | 0.00 | 0.00 | | 0.60 | 0.68 | 0.55 | 1.21 | 0.39 |
| *Syntrophorhabdus* | 0.00 | 0.00 | 0.00 | 0.00 | 0.00 | 0.00 | 0.00 | 0.00 | 0.00 | | 1.68 | 0.84 | 0.89 | 1.11 | 0.67 |
| *Syntrophus* | 0.00 | 0.00 | 0.00 | 0.00 | 0.00 | 0.00 | 0.00 | 0.00 | 0.01 | | 2.59 | 0.34 | 0.37 | 0.67 | 0.37 |
| *Smithella* | 0.00 | 0.00 | 0.00 | 0.00 | 0.00 | 0.00 | 0.00 | 0.00 | 0.00 | | 3.06 | 0.63 | 0.60 | 1.58 | 0.93 |
| *Petrotoga* | 0.00 | 0.00 | 0.00 | 0.06 | 0.20 | 0.27 | 1.36 | 0.99 | 0.56 | | 0.08 | 0.00 | 0.02 | 0.24 | 0.00 |
| *other* | 13.72 | 6.67 | 16.64 | 5.99 | 5.61 | 6.04 | 7.89 | 7.36 | 5.33 | | 19.02 | 29.11 | 23.21 | 18.81 | 31.16 |
| *unclassified* | 70.19 | 75.54 | 74.38 | 81.09 | 85.50 | 84.46 | 81.80 | 84.39 | 83.71 | | 64.87 | 59.75 | 67.52 | 58.76 | 61.50 |

|  | MM1 | MM2 | MM3 | MT1 | MT2a | MT2b | MT3a | MT3b | MT4 | SM1 | SM2 | SM3 | SM4 | SM5 |
| --- | --- | --- | --- | --- | --- | --- | --- | --- | --- | --- | --- | --- | --- | --- |
| EC:1.12.1.2 | 1.11E-04 | 1.74E-04 | 6.17E-04 | 6.64E-04 | 3.92E-04 | 3.93E-04 | 3.84E-04 | 7.28E-04 | 3.61E-04 | 0.0069 | 0.00448 | 0.00725 | 0.00646 | 0.00806 |
| EC:1.12.7.2 | 0.01002 | 0.01124 | 0.00812 | 0.00923 | 0.01082 | 0.01144 | 0.00566 | 0.00558 | 0.006 | 0.00749 | 0.00539 | 0.00392 | 0.00564 | 0.00369 |
| EC:1.12.98.1 | 0.00695 | 0.01381 | 0.00322 | 0.01897 | 0.00463 | 0.00465 | 0.02338 | 0.02588 | 0.02225 | 0.00482 | 0.00791 | 0.00495 | 0.00332 | 0.00451 |
| EC:1.12.98.2 | 6.28E-04 | 6.94E-05 | 2.40E-04 | 0 | 1.42E-04 | 1.43E-04 | 3.49E-05 | 0 | 0 | 1.59E-04 | 2.91E-04 | 7.07E-05 | 3.57E-05 | 0.00132 |
| EC:1.2.1.2 | 0.05353 | 0.06204 | 0.04165 | 0.03743 | 0.02998 | 0.02709 | 0.02166 | 0.01978 | 0.02189 | 0.05194 | 0.05418 | 0.06217 | 0.04225 | 0.05966 |
| EC:1.2.1.43 | 3.70E-05 | 0 | 0 | 0 | 0 | 0 | 0 | 0 | 0 | 1.20E-04 | 0 | 0 | 1.43E-04 | 1.43E-04 |
| EC:1.2.99.5 | 0.01501 | 0.01471 | 0.00764 | 0.0472 | 0.00431 | 0.00447 | 0.01908 | 0.02186 | 0.0289 | 0.00618 | 0.01796 | 0.01389 | 0.00803 | 0.02274 |
| EC:1.5.1.- | 3.70E-05 | 1.39E-04 | 0.00161 | 4.32E-04 | 5.34E-04 | 6.79E-04 | 2.80E-04 | 5.54E-04 | 2.17E-04 | 0.00634 | 0.00182 | 0.00304 | 0.00378 | 0.0015 |
| EC:1.5.99.11 | 0.00159 | 0.00933 | 0.0011 | 0.00701 | 0.00231 | 0.00197 | 0.01345 | 0.01299 | 0.01015 | 0.00498 | 0.00955 | 0.00912 | 0.00414 | 0.00906 |
| EC:1.5.99.9 | 8.87E-04 | 0.00656 | 3.08E-04 | 0.00535 | 0.00164 | 0.00204 | 0.01097 | 0.01261 | 0.00672 | 0.00319 | 0.00714 | 0.00541 | 0.00257 | 0.00598 |
| EC:2.3.1.101 | 0.00891 | 0.0085 | 0.00486 | 0.00757 | 0.00367 | 0.00347 | 0.01338 | 0.01341 | 0.01102 | 0.00427 | 0.00893 | 0.00735 | 0.00321 | 0.0097 |
| EC:3.5.4.27 | 0.00111 | 0.01416 | 0.0024 | 0.00621 | 0.0032 | 0.0035 | 0.01328 | 0.01524 | 0.00986 | 0.00442 | 0.00772 | 0.00622 | 0.00382 | 0.00795 |
| EC:4.3.-.- | 0 | 0 | 0 | 0 | 0 | 0 | 0 | 0 | 0 | 0 | 0 | 0 | 0 | 0 |
| EC:2.3.1.8 | 0.03006 | 0.01024 | 0.0312 | 0.01501 | 0.01118 | 0.01136 | 0.01192 | 0.01282 | 0.0104 | 0.03448 | 0.02652 | 0.02651 | 0.02759 | 0.03176 |
| EC:2.7.2.1 | 0.03771 | 0.0271 | 0.03322 | 0.03391 | 0.03724 | 0.03448 | 0.02708 | 0.02775 | 0.03197 | 0.02479 | 0.01716 | 0.01601 | 0.02127 | 0.01662 |
| EC:6.2.1.1 | 0.05915 | 0.00219 | 0.01856 | 0.00256 | 0.00217 | 0.00361 | 0.00992 | 0.01116 | 0.00199 | 0.06051 | 0.04835 | 0.06054 | 0.05317 | 0.06575 |
| ACDS | 0.06862 | 0.09355 | 0.04374 | 0.08161 | 0.03524 | 0.0338 | 0.05165 | 0.05415 | 0.04606 | 0.02404 | 0.04875 | 0.04156 | 0.02127 | 0.04602 |
| EC:1.1.1.272 | 0.00104 | 7.98E-04 | 0.00113 | 0.00116 | 5.70E-04 | 5.72E-04 | 5.24E-04 | 2.43E-04 | 8.31E-04 | 2.79E-04 | 4.01E-04 | 2.12E-04 | 5.71E-04 | 7.52E-04 |
| EC:3.1.3.71 | 9.61E-04 | 0.00163 | 0.00158 | 0.00355 | 0.00274 | 0.00314 | 0.00294 | 0.0034 | 0.00249 | 0.00247 | 0.00204 | 0.00205 | 0.00393 | 0.00254 |
| EC:4.1.1.79 | 7.39E-05 | 3.47E-05 | 2.74E-04 | 0.00316 | 0 | 7.15E-05 | 6.99E-05 | 1.04E-04 | 9.75E-04 | 6.38E-04 | 4.01E-04 | 0.00194 | 8.56E-04 | 0.00204 |
| EC:4.4.1.19 | 7.39E-04 | 0.00732 | 0.00301 | 0.01694 | 0.02407 | 0.02169 | 0.01387 | 0.01348 | 0.0224 | 0.00434 | 0.00357 | 0.00283 | 0.00382 | 0.00387 |
| MtaA | 4.81E-04 | 3.47E-05 | 2.74E-04 | 6.64E-05 | 0 | 7.15E-05 | 0 | 2.08E-04 | 0 | 1.20E-04 | 4.01E-04 | 4.59E-04 | 7.14E-05 | 5.01E-04 |
| MtaB | 8.13E-04 | 0 | 5.48E-04 | 0.00521 | 7.12E-05 | 0 | 1.05E-04 | 2.43E-04 | 0.00155 | 1.59E-04 | 1.09E-04 | 1.41E-04 | 7.14E-05 | 1.07E-04 |
| MtaC | 6.65E-04 | 6.94E-05 | 4.45E-04 | 0.00289 | 4.98E-04 | 6.43E-04 | 3.84E-04 | 5.20E-04 | 0.00137 | 3.19E-04 | 1.46E-04 | 2.83E-04 | 1.07E-04 | 2.51E-04 |
| EC:2.1.1.86 | 0.00832 | 0.03581 | 0.00534 | 0.03517 | 0.01061 | 0.01186 | 0.06199 | 0.06146 | 0.05282 | 0.01164 | 0.0286 | 0.02258 | 0.01406 | 0.02517 |
| EC:2.8.4.1 | 0.00462 | 0.00746 | 0.00257 | 0.0097 | 0.00256 | 0.00218 | 0.0145 | 0.01414 | 0.01286 | 0.00562 | 0.01177 | 0.00986 | 0.00614 | 0.01139 |
| EC:1.8.98.1 | 0.0095 | 0.01714 | 0.00764 | 0.01611 | 0.01218 | 0.01111 | 0.01981 | 0.01947 | 0.01503 | 0.0175 | 0.01818 | 0.02085 | 0.01524 | 0.01762 |

Table S3 Relative abundance of each gene involved in methanogenesis pathways from metagenomic datasets of the 14 samples

Table S4 Individual VFAs in each sample

| Sample name | Acetate (mM) | Propionate (mM) | Isobutyrate (mM) | Butyrate (mM) | Isovalerate (mM) | Valerate (mM) |
| --- | --- | --- | --- | --- | --- | --- |
| MM1 | 6.02±1.87 | 0.23±0.02 | 0.02±0 | 0 | 0.01±0.002 | 0.03±0.04 |
| MM2 | 6.74±0.52 | 0.12±0.05 | 0.02±0.01 | 0.01±0.001 | 0.02±0.005 | 0.04±0.03 |
| MM3 | 5.49±0.19 | 0.07±0.005 | 0.01±0.001 | 0 | 0.01±0.001 | 0 |
| MT1 | 1.89±0.44 | 0.04±0.01 | 0 | 0.01±0.001 | 0 | 0 |
| MT2a | 18.43±8.7 | 2.66±0.23 | 0.13±0.04 | 0.02±0.004 | 0.06±0.01 | 0 |
| MT2b | 9.68±2.31 | 0.97±0.01 | 0.04±0.001 | 0 | 0.02±0.001 | 0 |
| MT3a | 4.30±0.47 | 0.16±0.003 | 0.01±0.001 | 0 | 0.01±0.001 | 0.01±0.01 |
| MT3b | 1.02±0.19 | 0.02±0.003 | 0 | 0.01±0.001 | 0 | 0 |
| MT4 | 13.12±0.01 | 2.31±0.17 | 0.13±0.001 | 0.01±0.001 | 0.07±0.001 | 0.02±0.01 |
| SM1 | 1.15±0.13 | 0.03±0.003 | 0 | 0 | 0 | 0 |
| SM2 | 0.60±0.02 | 0.01±0.002 | 0 | 0 | 0 | 0 |
| SM3 | 0.61±0.07 | 0.01±0.001 | 0 | 0.02±0.001 | 0 | 0 |
| SM4 | 0.57±0.07 | 0.02±0.001 | 0 | 0.01±0.01 | 0 | 0 |
| SM5 | 1.67±1.31 | 0.01±0.01 | 0 | 0.01±0.005 | 0 | 0 |

Figure S1 Relative abundances of major categories of functional genes in the shotgun metagenomes obtained from the 14 samples.

Figure S2 Genes involved in carbohydrate and protein pathways from metagenomic datasets of the 14 samples

Figure S3 The ratio of ^14^CO_2_/^14^CH_4_ of the 14 samples

Figure S4 Correlation between the ratio of ^14^CO_2_/^14^CH_4_ and percentage of *Methanosarcinales*

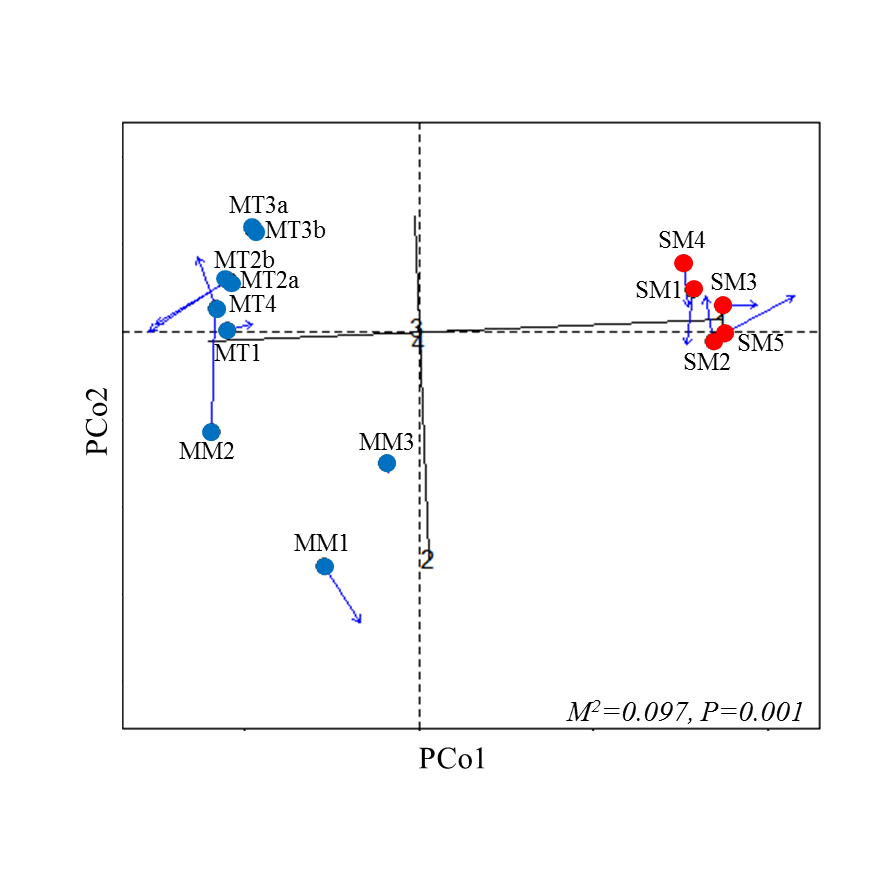


Figure S5 Procrustes analyses of taxonomic and functional patterns
